# Risk Mitigation for Immunocompromised Consumers of Mucormycete Spoiled and Fermented Foods: Germane Guidance and Remaining Needs

**DOI:** 10.3390/microorganisms6020045

**Published:** 2018-05-18

**Authors:** Abigail B. Snyder, Randy W. Worobo

**Affiliations:** 1 Department of Extension, The Ohio State University, Wooster, OH 44691, USA; 2 Department of Food Science, Cornell University, Ithaca, NY 14853, USA; rww8@cornell.edu

**Keywords:** fungal spoilage, mold fermented food, invasive fungal infections

## Abstract

Mucoralean invasive fungal infections, while unusual among the general population, have a high mortality rate among immunocompromised individuals who become infected. They are also common spoilage organisms in cultured dairy products, some fresh produce, and baked goods. Additionally, *Mucor* and *Rhizopus* spp. are utilized in the production of traditional fermented foods including mold ripened cheeses and fermented soy products. The risk that consumption of these foods poses to immunocompromised consumers has been previously identified. However, actionable guidance on implementation of appropriate dietary restrictions and microbial specification targets for food manufacturers serving these populations is scarce and is limited by insufficient data regarding traceback analysis in cases of invasive fungal infections where food is the suspected transmission vector. Culture-dependent and molecular subtyping methods, including whole genome sequencing, will improve identification of the point source. In turn, the empirically determined information on root-cause can best direct the development of appropriate food safety policies and programs.

## 1. The Proposed Risk of Foodborne Filamentous Fungi Causing Invasive Infections

In the fall of 2013, a Class II recall of Greek yogurt was issued due to contamination and spoilage from the dimorphic fungus, *Mucor circinelloides,* after consumers reported visible quality defects and symptoms of temporary gastroenteritis [1]. A single report of an *M. circinelloides* rhinocerebral infection in an immunocompromised individual who had consumed yogurt from the recalled lot was published [2]. Although the link between the case report and the spoiled product was not definitively confirmed with molecular subtyping methods, the risk of invasive filamentous fungal infections in immunocompromised individuals who consume foodborne fungi is an area of growing concern, particularly as the global incidence of immune suppression continues to rise [3].

This issue has been raised by Paterson and Lima (2017) and Benedict et al. (2016) [4,5]. However, these reports largely summarize (1) the prevalence of opportunistic fungal pathogens in food, beverages, and supplements and (2) case reports of invasive fungal infections that are potentially linked to food which, while providing a framework to justify additional studies, requires further evaluation of causation through traceback assessments in order to come to actionable conclusions. The attack rate for invasive fungal infections spread through food and water is unknown, and the significant factors influencing morbidity have not been described except through speculation and anecdotal reports. Generally speaking, the immunocompetent body experiences multitudinous exposure to fungal cells through air, food, and contact with fomites which does not result in disease manifestation. The innate and, to a lesser extent, adaptive immune systems sense and eliminate fungi from the lungs, skin, and gastrointestinal tract [6].

However, the incidence of invasive fungal infections increases drastically among immune suppressed populations—those with AIDS, diabetes, organ transplants, implanted medical devices, significant burns, and hematopoietic cell transplantation recipients. Subsequently, the host-specific factors regarding health status render an epidemiologically linked foodborne “outbreak” difficult to detect since only a highly specific subpopulation of consumers may ever experience disease [6]. The risk of invasive fungal infection through consumption of contaminated, spoiled, or mold fermented foods is likely based on a variety of factors, in addition to health status of the host. The intersection of host health-status, fungal load, and pathogenicity factors of the given fungal isolate all likely influence dose-response. Additionally, the ubiquitous presence of filamentous fungi in the environment can also confound linking the clinical isolate source to a food vector [6]. Without more explicit information regarding infection mechanisms and risk factors, risk assessments and, subsequently, policy development remain limited.

As an example of the potential food safety risk to immunocompromised individuals regarding consumption of foodborne filamentous fungi, mucoralean fungi represent a particularly pertinent class. Significant food spoilage and fermentation genera included in this class, *Mucor* and *Rhizopus* spp., have caused cutaneous, hematological, gastrointestinal, rhinocerebral, and sinopulmonary infections with a high probability of dissemination and mortality [3,7,8]. Across all types of sepsis, the mortality rate for mucormycosis is around 50%, with infection dissemination increasing mortality to 96% [9]. With the exception of a mycotoxin-mediated outbreak of 3-nitropropionic acid from *M. circinelloides* contaminated grains, the toxigenic potential of these basal fungi is putatively limited although, as with many toxin-mediated food safety hazards, it requires further investigation [10]. Other features of mucoralean secondary metabolism make them valuable in industrial fermentations and food production. *Mucor* and *Rhizopus* spp. are utilized in production of fermented soy and dairy products, and are also problematic spoilage organisms in the diary, grain, and produce industries [11].

The possible relationship between fungal food spoilage/fermentation and invasive fungal infections was described in a structured review by Benedict et al. (2016) wherein the authors queried case reports of fungal infections putatively linked to consumption of fermented or spoiled food, beverages, and dietary supplements [6]. Of the 11 reports, five were attributed to mucormycetes. However, many of the papers’ proposed putative causative relationships between foods and disease with varying degrees of evidence. For example, an incident of a patient presenting gastrointestinal distress three hours after consuming temperature abused food yielded a positive *M. indicus* culture in the patient’s blood and feces [12]. The rapid onset (indeed, Benedict et al. note the possibility for toxin mediation) and source of the infective pathogen are atypical of foodborne infections of the blood and feces and, without classical microbiology and molecular biology established links, the association between the temperature abused food and sepsis may be unrelated. In another incidence of *M. indicus* infection not included in the review, Deja et al. (2006) report a gastrointestinal infection wherein the patient “did not take any naturopathic medicine nor did he especially favor Asian food,” although the authors do note the use of *M. indicus* in the fermented products and the suggestion that, as a gastrointestinal infection, an oral route of infection may be suspected [13]. Similarly, two additional cases of immune suppressed individuals presenting gastrointestinal *Rhizopus* and *Mucor* infections were linked to patients’ consumption of home brewed beer and a fermented corn beverage (ngodwana), respectively [6,14,15]. In each of these cases, the link between foodborne fungi and invasive fungal infection is largely speculative.

These cases lack a concrete link to a contaminated product via culture dependent and/or molecular techniques. By comparison, a final example of a putatively food-linked case of mucormycosis occurred among 12 hematological oncology patients in a Hong Kong teaching hospital [16]. *R. microspores* was cultured from the blood and stool of five patients, and the stool of an additional seven (two with symptoms, five asymptomatic). Epidemiological and culture-based approaches were used to determine the source of the pathogen. A total of 706 samples (clinical, environmental, air, food, and drug) were cultured and *R. microspores* was isolated from chopsticks, allopurinol tablets, and from pre-packaged food. The authors claim that molecular subtyping showed that the clinical isolates were identical to both the strains from the drug and the pre-packaged food, but ITS1-ITS2 sequencing was used as the target amplicon and is not sensitive enough to prove point source. Indeed, the authors note an attempt to use RAPD (random amplification of polymorphic DNA) was performed with limited evidence of clonal distribution, underscoring the need for a generalizable method to subtype fungal isolates in traceback analysis.

Anecdotal or exclusively epidemiologically-based reports are limited by lack of traceback evaluations, which would provide solid evidence for a root cause. Morin (2016) notes that morphological typing is unreliable, and, in most clinical cases, isolate identification is not performed, or the result is often reported to the genus-level only [3,17]. Without more concrete information on the identity of the isolate, outbreak clades cannot be established. Indeed, the whole genome sequencing of 21 isolates, collected during an outbreak of invasive wound mucormycosis within two burn units, was recently published and is reportedly the first incidence of whole genome sequencing (WGS) utilization in such a case [18]. Crucially, the authors initially believed a single environmental source was responsible for the infections among patients within a given burn unit; however, that theory was revised when the diversity of the clinical isolate strains was revealed through WGS. The authors instead concluded that it was more likely that patients were infected from a pool of diverse strains from the environment rather than from a shared source, which indicates the complexity of determining the source of mucoralean fungi implicated in invasive infections among immunocompromised individuals [18]. This lack of available root-cause evidence inherently limits the development of policies and best practices for food safety recommendations for immunocompromised consumers, the manufacturers of their food, and their health-care providers.

## 2. Guidance and Regulations Regarding Fungi in Food

There are minimal food safety regulatory restrictions regarding fungal spoilage and fermented products in the U.S.—the perceived risk due largely to mycotoxin chemical hazards as opposed to invasive fungal infections transmitted via food. In most cases, visible fungal growth is used as a proxy for potential mycotoxin production risk and, outside products in which this is an identified hazard, fungal growth is principally perceived as a quality defect, indicator of poor sanitation, or undesirable condition(s). Perhaps the most comprehensive example of this is the FDA’s Defect Action Levels, which fall under Good Manufacturing Practices (GMPs) in 21 CFR Part 117.110, and are published limits established on the premise that natural or unavoidable defects pose no inherent hazard to health. Generally, these include defects such as insects, larvae, or insect fragments, rodent filth, mold, shell fragments, and stems, among others. While the presence of these defects is not considered directly hazardous, excessive levels violates standards set forth in GMPs and the product is termed adulterated. However, producing food products totally devoid of these defects is considered impractical and economically non-viable as many of the defects occur naturally or are regarded as unavoidable [19].

Notably, products that are within these parameters can still be subject to regulatory action if they are determined to be harmful to consumers, and manufacturers are encouraged to recognize these levels as extreme limits rather than typical maxima for day-to-day production [19]. If a defect action level does not exist for a product, FDA can evaluate defects on a case-by-case basis considering various criteria related to filth and extraneous material—length of hairs, size of insect fragments, distribution in the sample—to determine if a product should be considered adulterated. Products can be adulterated if they are considered “moldy,” an assessment based on evidence of the presence of mold (hyphae) visible to the unaided eye, although microscopy can be used to confirm typical characteristics. Defect action levels can also be based on mold count using the Howard method in which the percentage of positive microscopic fields that contain hyphae are determined. This method is used in processed material where hyphae may no longer be visible due to the comminution process [19]. The defect action levels established for filamentous fungal growth in foods are excerpted in Table 1, below.

Defect action levels represent minimum standards for sanitation, rather than empirically determined controls for safety. Allergenic response and invasive fungal infections are not typically identified as hazards in food safety plan development, though the consumer facing guidance from the USDA as well as food retail guidance includes concrete, reactive responses to food spoiled by mold growth. This includes removal of fungal growth with an additional 1-inch radius around damaged areas in select foods—hard cheeses, hard meats, and firm vegetables (e.g., carrots, peppers) [20]. Discard is recommended among other foods with visible mold growth due to mycotoxin secretion and diffusion, hyphal penetration beyond the visible surface, and co-colonization of the food with fungi and bacteria [21].

For food manufacturers supplying institutions and high-risk populations, more restrictive microbiological standards and policies may be implemented. Neutropenic diets may variably be recommended for immunocompromised patients, but the efficacy of these programs in reducing the incidence of infection complications is not well established [2,6]. Products like infant formula, drugs and medical devices, and food produced for hospitals and nursing homes may have increased regulatory or company-specific standards applied, given the immune status of the consumer. However, even with the increased oversight over food safety, many times this implies increased efforts (finished product testing, increased monitoring frequency, more conservative critical limits) regarding the identified bacterial pathogens, rather than total aerobic count or fungal load. Guidelines published by Tomblyn et al. [22] for preventing infections among hematopoetic stem cell transplant recipients and were co-sponsored by the Centers for Disease Control and Prevention [22]. The food safety guidelines largely focused on eliminating raw and many fermented foods from the diet. Specifically, raw products—including raw grains, raw honey, and certain raw produce that could not be washed and peeled—mate tea (steeped yerba mate leaves), and herbal and nutritional supplements were included. Fermented products including hard cheeses with mold (Blue, Stilton, Roquefort, Gorgonzola), tempeh, all miso products, products with uncooked brewer’s yeast, and hard cured salami in natural wrap were explicitly mentioned [22]. Avoidance of food products with potentially high doses of opportunistic fungi is a principle focus of this guidance.

## 3. Incidence of Mucormycetes in Foods

As indicated by the exclusion of several mucoralean fermented products described in the list above, concerns regarding the transmission of invasive fungal infections through foods among high-risk populations remains a concern in clinical settings. Notably, some opportunistic fungi which cannot colonize the mucosa of the gastrointestinal tract can cause severe infections of the respiratory system [23,24]. Fungal gastrointestinal infections can come from ingestion, but may also originate in the lungs and disseminate, so it is difficult to exclude this possibility and conclusions that infections of the gastrointestinal tract necessarily originated from a food source may be spurious [6]. Fungal load on food products is also likely a contributing factor in evaluating consumer risk. *Mucor* and *Rhizopus* spp. are readily present in the environment, and several studies report isolation from various foods including produce, bread, and cheese [4,25,26]. However, detection alone from within the background biota of a food product represents a potentially lower degree of exposure than would consumption of a spoiled or mold fermented food where cellular counts have increased over several orders of magnitude.

Mucormycetes may be introduced and propagate during processing and shelf-life, or they may be intrinsically present in certain products [6,11]. They are perhaps most notoriously associated with spoilage of cultured dairy products including cream cheese, cheese, and yogurt. On some cheeses, proliferation of the aerial hyphae characterizes the so-called “cat-hair” defect on the cheese surface [10]. *Mucor* and *Rhizopus* spp. also cause post-harvest spoilage in produce, including pome fruit (apples and pears), berries, tomatoes, eggplant, various stone fruits (cherries, peaches, etc.), root vegetables (onion and potatoes) and cabbage, as well as in grains, cereals, baked goods, raisins, salami, and other low to medium water activity products [25,27,28].

Perhaps the product category of most concern regarding mucormycete transmission through food is that of mold fermented products which rely on *Mucor* and/or *Rhizopus* in production. Although mucormycetes sometimes cause spoilage in cheeses, in certain varieties, the enzymatic activity of *Mucor* spp. contributes desirable organoleptic properties due to their secretion of lipolytic and proteolytic enzymes during ripening [27]. These varieties include Saint Nectaire, Tomme de Savoie, and Taleggio [10]. In fact, a survey of >50 mold ripened cheese varieties determined that *Mucor* was the second most prominent genus, following penicillia, found in 27% of the cheeses in the study [29,30]. Mucormycetes are also utilized in Asian and African fermented grain products. These include sufu (fermented tofu), ragi (fermented, ground millet), tempeh (fermented cooked soybeans), furu (fermented bean curd/tofu), and douchi (fermented soybean) [3]. Several varieties of douchi are produced depending on the fermentation culture, two of which are *Mucor*-type and *Rhizopus*-type [31].

There is some debate about the differences in infective potential among select fermentation strains, those associated with spoilage, and those from clinical specimens. Among some 58 known *Mucor* spp., there are several major players in each of these three functions. For example, *M. circinelloides* is a common spoilage organism but is also frequently identified as an invasive fungal pathogen [3]. *R. oryzae* is the etiological agent in 90% of the rhinocerebral cases of mucormycosis, and is the same species utilized in tempeh production [2]. Utilizing species-level identification as an aid to assess if a clinical specimen is likely from a food source may be premature. Morin (2016) cautions that these suppositions may be inaccurate given the frequency of misidentification to the species level. Although species identification is sometimes referenced to indicate an isolate is primarily associated with one of these three categories, it is likely that the taxa has not been sufficiently resolved to utilize such conclusions [1,3,10].

## 4. Conclusions

The risk for invasive fungal infections transmitted through food is poorly characterized but is greater among immune suppressed individuals. As the global population of immune suppressed individuals increases, and our ability to perform molecular traceback analysis in filamentous fungi isolated from food and clinical specimens likewise increases, we need consider the development of actionable policies and guidelines regarding dietary restrictions for the individual (e.g., avoiding certain fermented products), microbiological criteria and specifications for manufacturers (e.g., total aerobic count, yeast and mold, and/or specific fungal genera detection), and potentially enforceable risk-based regulations geared toward safety rather than quality attributes extending from filamentous fungi growth in food. However, the needless development of microbiological criteria for its own sake should be avoided. Therefore, proposal of policies, guidance, best-practices, and regulations must be risk-based and developed from causal outbreaks and not anecdotal cases.

## Figures and Tables

**Table 1:** Defect action levels for filamentous fungal growth in various food products as reported in the U.S. FDA Defect Levels Handbook. Information excerpted from [19].

Product	Defect	Action Level	Source
Allspice, whole	Mold	Average of 5% or more berries by weight are moldy	Significance—potential health hazard-may contain mycotoxin producing fungi
Apple Butter	Mold	Average of mold count is 12% or more	Post-harvest infection. Significance—aesthetic
Bay (Laurel) Leaves	Mold	Average of 5% or more pieces by weight are moldy	Pre-harvest infection. Significance—aesthetic
Beets, Canned	Rot	Average of 5% or more pieces by weight with dry rot	Pre-harvest mold infection. Significance—aesthetic
Berries: Drupelet, Canned and Frozen	Mold	Average mold count is 60% or more	Post-harvest infection. Significance—aesthetic
Capsicum Pods	Mold	Average of more than 3% of pods be weight are moldy	Potential health hazard—mold may contain mycotoxin producing fungi
Ground Capsicum (excluding paprika)	Mold	Average mold count is more than 20%	Pre-harvest mold infection. Significance—mold may contain mycotoxin producing fungi
Ground Paprika	Mold	Average mold count is more than 20%	Potential health hazard—mold may contain mycotoxin producing fungi
Cassia or Cinnamon Bark, whole	Mold	Average of 5% or more pieces by weight are moldy	Post-harvest infection. Significance—aesthetic
Cherries: Fresh, Canned, or Frozen	Rot	Average of 7% or more pieces are rejects due to rot	Rot reject—pre-harvest mold infection. Significance—aesthetic
Cherry Jam	Mold	Average mold count is 30% or more	Pre-harvest mold infection. Significance—aesthetic
Citrus Fruit Juices, Canned	Mold	Average mold count is 10% or more	Processing contamination. Significance—aesthetic
Cocoa Beans	Mold	More than 4% of beans by count are moldy	Post-harvest infection. Significance—potential health hazard—may contain mycotoxin producing fungi
Coffee Beans, Green	Mold	Average of 10% or more beans by count are moldy	Post-harvest infection. Significance—potential health hazard—may contain mycotoxin producing fungi
Corn Husks for Tamales	Mold	Average of 5% or more husks by weight are moldy	Pre-harvest and/or post-harvest and/or processing infection. Significance—aesthetic
Cranberry Sauce	Mold	Average mold count is more than 15% OR the mold count of any one subsample is more than 50%	Pre-harvest and/or post-harvest infection. Significance—aesthetic
Current Jam, Black	Mold	Average mold count is 75% or more	Post-harvest and/or processing infection. Significance—aesthetic
Greens, canned	Mildew	Average of 10% or more of leaves, by count or weight, showing mildew over 1/2″ in diameter	Pre-harvest infection. Significance—aesthetic
Nectars, Apricot, Peaches, and Pear	Mold	Average mold count is 12% or more	Pre-harvest infection. Significance—aesthetic
Tree nuts	Moldy	Between 5% and 15% as determined by macroscopic examination depending on type and un/shelled	Pre-harvest and/or post-harvest and/or processing infection. Significance—potential health hazard—may contain mycotoxin producing fungi
Salt-Cured Olives	Mold	Average of 25% or more olives by count are moldy	Post-harvest and/or processing infection. Significance—aesthetic
Peaches, Canned and Frozen	Mold	Average of 3% or more fruit by count are moldy	Pre-harvest and/or post-harvest infection. Significance—aesthetic
Peanuts, Shelled Peanuts, Unshelled	Moldy	Average of 5% or more kernels by count are rejects; Average of 10% or more kernels by count are rejects	Pre-harvest and/or post-harvest and/or processing infection. Significance—potential health hazard—may contain mycotoxin producing fungi
Pepper, Whole	Mold	Average of 1% or more pieces by weight are moldy	Post-harvest and/or processing infection. Significance—aesthetic
Pineapple, Canned Pineapple Juice	Mold	Average mold count is 20% or more OR the mold count of any one sample is 60% or more; Average mold count is 15% or more OR the mold count of any one sample is 40% or more	Processing. Significance—aesthetic
Plums, Canned	Rot	Average of 5% or more plums by count with rot spots larger than the area of a circle 12 mm in diameter	Pre-harvest and/or post-harvest infection. Significance—aesthetic
Potato Chips	Rot	Average of 6% or more pieces by weight contain rot	Pre-harvest and/or post-harvest infection. Significance—aesthetic
Prunes, Dried and Dehydrated, Low-Moisture	Moldy	Average of a minimum of 10 subsamples in 5% or more prunes by count are rejects	Pre-harvest infection. Significance—aesthetic
Puree, Apricot, Peach, and Pear	Mold	Average mold count is 12% or more	Pre-harvest and/or post-harvest and/or processing infection. Significance—aesthetic
Raisins, Natural & Golden	Mold	Average of 10 subsamples is 5% or more by count, moldy raisins	Post-harvest and/or processing infection. Significance—aesthetic
Sesame Seeds	Mold	Average of 5% or more seeds by weight are decomposed	Pre-harvest infection. Significance—aesthetic
Strawberries: Frozen Whole or Sliced	Mold	Average mold count or 45% or more and mold count of at least half of the subsamples is 55% or more	Post-harvest and/or processing infection. Significance—aesthetic
Tomatoes, Canned with or without Juice (based on drained juice); Tomatoes, Canned Packed in Tomato Puree (based on drained liquid)	Mold	Average mold count in 6 samples is 15% or more and the counts of all of the subsamples are more than 12%; Average in 6 subsamples is 29% or more and the counts of all of the subsamples are more than 25%	Pre-harvest and/or post-harvest and/or processing infection. Significance—aesthetic
Tomato Juice	Mold	Average mold count in 6 subsamples is 24% or more and the counts of all of the subsamples are more than 20%	Pre-harvest and/or post-harvest and/or processing infection. Significance—aesthetic
Tomato Paste or Puree; Tomato Sauce, Undiluted; Tomato Powder, Except Spray-Dried; Tomato Soup and Tomato Products	Mold	Average mold count in 6 samples is 45% or more and the mold counts of all of the subsamples are more than 40%	Pre-harvest and/or post-harvest and/or processing. Significance—aesthetic
Pizza and other Tomato Sauces	Mold	Average mold count in 6 samples is 34% or more and the mold counts of all of the subsamples are more than 30%	Pre-harvest and/or post-harvest and/or processing. Significance—aesthetic
Tomato Catsup	Mold	Average mold count in 6 samples is 55% or more	Pre-harvest and/or post-harvest and/or processing. Significance—aesthetic
Tomato Powder, Spray-Dried	Mold	Average mold count in 6 subsamples is 67% or more	Pre-harvest and/or post-harvest and/or processing. Significance—aesthetic

## References

[1] Snyder A.B., Churey J.J., Worobo R.W. (2016). Characterization and control of *Mucor circinelloides* spoilage in yogurt. Int. J. Food Microbiol..

[2] Lazar S.P., Lukaszewicz J.M., Persad K.A., Reinhardt J.F. (2014). Rhinocerebral *Mucor circinelloides* infection in immunocompromised patient following yogurt ingestion. Deleware Med. J..

[3] Morin-Sardin S., Nodet P., Coton E., Jany J. (2016). *Mucor*: A Janus-faced fungal genus with human health impact and industrial applications. Fungal Biol. Rev..

[4] Paterson R.R.M., Lima N. (2017). Filamentous fungal human pathogens from food emphasizing *Aspergillus, Fusarium* and *Mucor*. Microorganisms.

[5] Benedict K., Chiller T.M., Mody R.K. (2016). Invasive fungal infections acquired from contaminated food or nutritional supplements: A review of the literature. Foodborne Pathog. Dis..

[6] Lionakis M.S., Levitz M.S. (2017). Host control of fungal infections: Lessons from basic studies and human cohorts. Ann. Rev. Immunol..

[7] Dizbay M., Adisen E., Kustimur S., Sari N., Cengiz B., Yalcin B., Kallkanci A., Gonul I.I., Sugita T. (2009). Fungemia and cutaneous Zygomycosis due to *Mucor circinelloides* in an intensive care unit patient: Case report and review of the literature. Jpn. J. Infect. Dis..

[8] De Repentigny L., St-Germain G., Charest H., Kokta V., Vobecky S. (2008). Fatal zygomycosis caused by *Mucor indicus* in a child with an implantable left ventricular assist device. Pediatr. Infect. Dis. J..

[9] Center for Disease Control and Prevention (2015). Mucormycosis. https://www.cdc.gov/fungal/diseases/mucormycosis/index.html.

[10] Hermet A., Meheust D., Mounier J., Barbier G., Jany J. (2012). Molecular systematics in the genus *Mucor* with special regards to species encountered in cheese. Fungal Biol..

[11] Snyder A.B., Worobo R.W. (2018). Fungal spoilage in food processing. J. Food Prot..

[12] Aboltins C.A., Pratt W.A., Solano T.R. (2006). Fungemia secondary to gastrointestinal *Mucor indicus* infection. Clin. Infect. Dis..

[13] Deja M., Wolf S., Weber-Carstens S., Lehmann T., Adler A., Ruhnke M., Tintelnot K. (2006). Gastrointestinal zygomycosis caused by *Mucor indicus* in a patient with acute traumatic brain injury. Med. Mycol. J..

[14] Martinello M., Nelson A., Bignold L., Shaw D. (2012). We are what we eat! Invasive intestinal mucormycosis: A case report and review of the literature.. Med. Mycol. Case Rep..

[15] Sutherland J.C., Jones T.H. (1960). Gastric mucormycosis: Report of case in Swazi. S. Afr. Med. J..

[16] Cheng V.C., Chan J.F.W., Ngan A.H., To K.K.W., Leung S.Y., Tsoi H.W., Yam W.C., Tai J.W.M., Wong S.S.Y., Tse H. (2009). Outbreak of intestinal infection due to *Rhizopus microsporus*. J. Clin. Microbiol..

[17] Skiada A., Pagano L., Groll A., Zimmerli S., Dupont B., Lagrou K., Lass Florl C., Bouza E., Klimko N., Gaustad P. (2011). Zygomycosis in Europe: Analysis of 230 cases accrued by the registry of the European Confederation of Medical Mycology (ECMM) Working Group on Zygomycosis between 2005 and 2007. Clin. Microbiol. Infect..

[18] Garcia-Hermoso D., Criscuolo A., Lee S.C., Legrand M., Chaouat M., Denis B., Lafaurie M., Rouveau M., Soler C., Schaal J.V. (2018). Outbreak of invasive wound Mucormycosis in a burn unit due to multiple strains of *Mucor circinelloides*
*f circinelloides* resolvd by whole-genome sequencing.. mBio.

[19] U.S. Food and Drug Administration (1995). Defect Levels Handbook. https://www.fda.gov/Food/GuidanceRegulation/GuidanceDocumentsRegulatoryInformation/SanitationTransportation/ucm056174.htm.

[20] National Restaurant Association ServSafe Food Protection Manager Training, Chapter 2 Forms of Contamination. https://www.servsafe.com.

[21] U.S. Department of Agriculture (2013). Molds on Food: Are They Dangerous?. https://www.fsis.usda.gov/wps/portal/fsis/topics/food-safety-education/get-answers/food-safety-fact-sheets/safe-food-handling/molds-on-food-are-they-dangerous_/ct_index.

[22] Tomblyn M., Chiller T., Einsele H., Gress R., Sepkowitz K., Storek J., Wingard J.R., Young J.H., Boekh M.A. (2009). Guidelines for preventing infectious complications among hematopoietic cell transplantation recipients: A global perspective. J. Am. Soc. Blood Marrow Transplant..

[23] Illiev I.D., Leonardi I. (2017). Fungal dysbiosis: Immunity and interactions at mucosal barriers. Nat. Rev. Immunol..

[24] David L.A., Maurice C.F., Carmody R.N., Gootenberg D.B., Button J.E., Wolfe B.E., Ling A.V., Devlin A.S., Varma Y., Fischbach M.A. (2014). Diet rapidly and reproducibly alters the human gut microbiome. Nature.

[25] Anwer S.S., Ali G.A., Hamadamin C.Z., Jaafar H.Y. (2017). Isolation and identification of fungi from fast food restaurants in Langa Bazar. Int. J. Environ. Agric. Biotechnol..

[26] Buehler A.J., Evanowski R.L., Martin N.H., Boor K.J., Wiedmann M. (2017). Internal transcribed spacer (ITS) sequencing reveals considerable fungal diversity in dairy products. J. Dairy Sci..

[27] Morin-Sardin S., Rigalma K., Coroller L., Jany J., Coton E. (2015). Effect of temperature, pH, and water activity on *Mucor* spp. growth on synthetic medium, cheese analog, and cheese. Food Microbiol..

[28] Snyder A.B., Churey J.J., Worobo R.W. (2018).

[29] Barrios M.J., Medina L.M., López M.C., Jordano R. (1998). Fungal biota isolated from Spanish cheeses. J. Food Saf..

[30] Fox P.F., McSweeney P.L.H., Fox P.F., McSweeney P.L.H., Cogan T.M., Guinee T.P. (2004). Cheese: An overview. Cheese: Chemistry, Physics and Microbiology.

[31] He G., Huang J., Liang R., Wu C., Zhou R. (2016). Comparing the differences of characteristic flavor between natural maturation and starter culture for *Mucor*-type *Douchi*. Int. J. Food Sci. Technol..

